# Non-linearity, complexity, and quantization concepts in biology

**DOI:** 10.3389/fnhum.2025.1695510

**Published:** 2026-01-07

**Authors:** Neil D. Theise, Jack A. Tuszynski

**Affiliations:** 1 Department of Pathology, NYU Grossman School of Medicine, New York, NY, United States; 2 Department of Mechanical and Aerospace Engineering (DIMEAS), Polytechnic University of Turin, Turin, Italy; 3 Department of Physics, University of Alberta, Edmonton, AB, Canada; 4 Allen Discovery Center, Tufts University, Medford, MA, United States

**Keywords:** biological coherence, Biological Uncertainty, complexity, Method of Coherent Structures, quantum biology, systems biology

## Abstract

Founders of quantum mechanics (QM) anticipated that revisions to classical physics due to strange elements of quantum reality, would necessitate similar changes in biology. Complexity theory, systems biology and quantum biology provide possible solutions indicating that subject/object separation is a useful fiction for reductive science. Direct correlates to such QM observational/measurement issues as Complementarity and Uncertainty may justify the introduction of an analog of Heisenberg's uncertainty and the Planck constant for living systems. The phase space of “adjacent possibles” for biological systems from which one “actual” is selected resembles the collapse of the QM wave function. Since biological systems are hierarchical, this occurs across organizational scales resulting in biological coherence. The location of a quantum/classical boundary is unclear due to complexity. Whether biological systems' characteristics arise directly from QM or are of a different origin remains unsettled. To combine non-linear with quantum properties across biological scales we propose the Method of Coherent Structures (MCS), developed for quantum many-body systems. In MCS, a higher-level scale provides a classical envelope for quantum fluctuations at the lower scale. It yields a seamless transition from non-linear classical fields to quantum excitations and accounts for the emergence of complexity by incorporating metabolic energy supply.

## Introduction

The question of the role quantum mechanics (QM) might play in biology was first raised by its founders. Niels Bohr wrote:

“*Thus the existence of life itself would have to be regarded in biology, both as regards the possibilities of observation and of definition, as no more subject to analysis than the existence of the quantum of action in atomic physics.”* (Bohr, 1937)

Erwin Schrödinger asserted that:

“*living matter, while not eluding the ‘laws of physics' as established up to date, is likely to involve ‘other laws of physics' hitherto unknown, which however, once they have been revealed, will form just as integral a part of science as the former.”* (Schrödinger, 1944)

Wolfgang Pauli, commenting on “the measurement problem,” wrote:

“*The precondition for a description of phenomena independently of the mode of their observation is no longer fulfilled, and physical objects acquire a two-valued, or many-valued, and therefore symbolic character*.” (Enz and von Meyenn, 1994)

For Pauli, this would include systems that were alive (Van Speybroeck, 2009).

While straightforward extensions of QM to chemical bonds and chemical reactions have been used to properly describe the behavior of chemical molecules using such formalisms as Dirac-Hartree-Fock or density functional methods (McQuarrie, 2008), quantum physics applications to biology are less obvious and merit close scrutiny, which this article aims to provide. All chemistry including biochemistry is based on the creation and destruction of bonds between atoms and molecules and hence on quantum interactions, so living systems, similarly to non-living systems, depend on quantum states at the level of fundamental building blocks. However, the unitary oneness and functional synchronization exhibited by living systems suggest that higher level quantum properties such as Bose-Einstein condensation, quantum coherent superposition and entanglement may also operate in biology. Also, the quantum principle of complementarity has been proposed to operate, for example, within neuroscience (Roy and Kafatos, 1999, 2004). However, quantum effects are expected to be washed out by thermal decoherence at scales larger than individual macromolecules or macromolecular complexes and in aqueous media. Thus, the likelihood of quantum states playing functional roles at mesoscopic or macroscopic scales in “warm, wet and noisy” biological systems has been seen as a serious impediment to quantum biology (Tegmark, 2000) However, this issue may not be as serious as first believed (Hagan et al., 2002) since various mitigating effects can be found such as shielding of quantum states by the ordering of water dipoles, and the presence of far-from thermal equilibrium states of living systems due to constant metabolic energy supply (Davis et al., 2010). Moreover, evolution through billions of years of experimentation and countless attempts of trial and error may have solved this problem resulting in the emergence of mesoscopic or even macroscopic quantum states in biological systems. If organized quantum states can exist in living cells they may possibly be integrated among components, organelles or even organs (especially the brain).

Quantum effects—such as quantum tunneling (Masgrau et al., 2006), superposition, wave-particle duality, entanglement, and coherence—are presumed to be essential for the normative functioning of complex biological systems and their disturbance in disease states such as cancer may provide new perspectives on both diagnostics and therapeutics (Davies et al., 2012). Hence, “quantum biology” has recently become a pioneering field of inquiry (McFadden and Al-Khalili, 2018; Feng et al., 2022). It is worth noting that in the early days of QM, only several examples of non-classical behavior were sufficient to undermine the supremacy of classical physics. A similar situation appears to exist today in biology with the mechanisms of photosynthesis (Collini et al., 2010; Engel et al., 2007), bird navigation (Ritz et al., 2004), olfaction, the sense of vision and the highly disputed field of human consciousness (Atmanspacher, 2004) being the main examples of quantum effects in living system.

More recently, the experiments (Kalra et al., 2023) performed on tubulin and microtubules showed that quantum excitations exhibited by tryptophan residues in these proteins and protein polymers generated by ultraviolet electromagnetic waves can persist for up to 5 ns and have a spatial coherence domain of up to 6 nm, roughly corresponding to the size of tubulin. Moreover, the presence of anesthetic molecules bound to tubulin shortens the lifetime of these quantum states providing an indirect connection to consciousness. These findings indicate feasibility of quantum effects in the microtubule cytoskeleton under laboratory conditions. Additional evidence of similar effects in actin and vimentin has just been reported (Kakati et al., 2024), which indicates a potential for cell-level quantization. Nonetheless, further experimental evidence for the existence of quantum states in cells, especially neurons, is required.

A major aspect of living systems that has resisted explanation using classical physics is coherence across the hierarchies of their organization. To address this problem H. Fröhlich, considered quantum interactions between membrane dipoles assisted by energy pumping provided by metabolic processes in the presence of thermal noise, and hypothesized the emergence of Bose-Einstein condensation of electromagnetic modes in the microwave range, propagating across the cell and coupling with other cells of the same type (Fröhlich, 1968). In the past few years, experimental evidence for this effect has been accumulating for various proteins as model systems (Lundholm et al., 2015; Nardecchia et al., 2018). While Bose-Einstein condensates are normally associated with very low-temperatures, Fröhlich showed that non-linear coupling between a collection of dipole oscillators in the cellular membrane driven by sufficiently high energy supply could indeed channel this energy into a single coherent dipolar oscillator even at biological temperatures. Recently, there has been renewed interest and some experimental support, for the Fröhlich condensate idea, which could play a dramatic role in chemical kinetics of far-from-equilibrium biological nano-systems. An application of this concept to the dipolar modes of neuronal membrane's head groups driven by action potentials has provided parameter estimates that generally agree with the Fröhlich hypothesis (Cavaglia and Tuszynski, 2025). This result additionally provides a potential connection to a holographic image formation (Cavaglià et al., 2023) proposal put forward by Pribram decades ago (Pribram, 1989). Furthermore, recent analysis of NMR data has revealed quantum hallmarks of the functioning of the human brain (Kerskens and Pérez, 2022).

Another unresolved and possibly related issue in biology is the high efficiency of energy transport at a cellular and molecular levels (Perez-Martin et al., 2024). Davydov (1982) proposed a quantum mechanical model involving a strong interaction between phonons and excitons in alpha helical structures such as those found in DNA and proteins. This model resulted in the formation of a non-linear lossless energy-carrying wave, called a Davydov soliton (Scott, 1992).

What was perhaps missing for these QM founders to fully explore biology, but has gradually developed over the next decades through the work of Davydov, Fröhlich, Haken, Prigogine and others, was a mathematical framework that could consistently model biological systems. The various mathematical tools that helped the 20th century physicists to codify the rules of QM (e.g., Schrödinger's wavefunction and Heisenberg's matrix mechanics) as well as relativity (e.g., non-Euclidean geometries, tensor calculus, Dirac's relativistic QM), appear inadequate for living systems. It was not until the developments of chaos theory, fractal mathematics, non-linear dynamics and, eventually, complexity theory, that mathematics could capture the non-linear dynamical nature of life. It is through the lens of such mathematical approaches that we can begin to explore the founders' intuited affinities between the seemingly strange implications of QM and those yet to be discovered in biology. These biological correlates of quantum “weirdness” come fully into view when one considers all scales of biology, from biomolecules to cells, to tissues, organs, and organisms, to systems of living beings in communities, cultures, societies and ecosystems.

## A complexity primer

Complexity theory concerns the nature of systems of interacting individuals or “agents” which tend to self-organize into self-perpetuating, adaptive structures (Theise, 2006; Theise and D'Inverno, 2004; Theise and Harris, 2006; Theise and Kafatos, 2013; Theise, 2023). These structures possess “emergent properties” and exhibit “emergence.” Biological systems are emblematic of complexity. A human cell has a DNA containing billions of base pairs with approximately 20,000 genes encoding the same number of distinct kinds of proteins. Cells respond to stimuli by activating vast numbers of protein-protein interaction networks. An organism has hundreds of types of cells and the human body is composed of some 36 trillion cells. The human brain is a collection of roughly 100 billion neurons and a similar number of glial cells. Each neuron interacts on average with as many as 10,000 other neurons via synaptic connections. Contrary to early naïve viewpoints, a cell, especially a neuron, is not a binary switch but a complex information processing unit (Albrecht-Buehler, 1992). The above numbers not only speak to the sheer complexity of the biological systems but also pose a conceptual challenge of understanding the hierarchical functional orchestration across over twenty orders of magnitude on the time scale (ranging from picoseconds for molecular vibrations to billions of seconds for lifetimes), and over twelve orders of magnitude on the spatial scale (from angstroms for atomic dimensions to meters for organismic sizes).

A system may be deemed complex when the interactions between its components fulfill some basic criteria summarized below (Theise, 2023):

- Numerical sufficiency. There must be sufficiently many elements interacting to generate emergent properties, though how many is sufficient depends on the nature of the agents involved. The more agents there are, the more complex the emergent properties may become and the greater the diversity of interactions leading to yet greater complexity.
- Predominance of negative over positive homeostatic feedback. Negative feedback loops keep interactions within homeostatic zones that promote sustainability and facilitate healthy adaptation. Positive feedback loops may be present and serve useful purposes in the near term, but they cannot predominate. If they do, these are likely to be energy expending and self-limited, rather than adaptive. Both positive and negative feedback loops are characteristic features of non-linear systems studied in pure mathematics (Fuchs, 2014) and in mathematical biology (Murray, 2007).
- All interactions are local. There is no global sensing. Interactions are local between individual interacting agents. While top-down influences may be present, the agents at the “top” are as influenced by those at the “bottom” and *vice versa*. In fact, there is no actual “top” or “bottom”—complex systems are multi-nodal (scale free) webs that are interconnected locally through multidirectional interactions, i.e., holarchies (Koestler, 1967). Cellular automata (Wolfram, 1983) offer a simple yet powerful example of models generating complex behavior. However, it may not be possible to explain all biological behaviors by invoking strictly local, spatial interactions. The evidence for bystander effects indicates long-distance interactions between cells and between organisms (Mothersill et al., 2018), which may even involve quantum entanglement (Matarèse et al., 2023).
- Limited randomness (or “quenched disorder”). If there is too much randomness in a system (compared to deterministic interactions), then there is no capacity for sustained self-organization and emergence, but if there is too little, then the system behaves more like a machine and loses the capacity to change its organizational structures in response to a changing environment. When the environment changes drastically, the failure to adapt will lead to a mass extinction event. This low-level randomness underlies the abilities of a system to explore what has been termed “the adjacent possibles” (Matarèse et al., 2023; Kauffman, 1995, 2019).

For example, all life forms exist at ambient temperatures and hence thermal noise contributes to an inevitable presence of randomness in all living processes. Each degree of freedom in a biological system experiences random fluctuations corresponding to the thermal energy of k_B_T/2 (approximately 0.6 kcal/mol) where k_B_ is the Boltzmann constant and T temperature in kelvin. The presence of thermal noise causing decoherence has been one of the persistent arguments against the use of quantum physics in biology. However, there exist physical systems that exhibit quantum characteristics at relatively high temperatures (lasers, magnets, phonons in crystal lattices and high-temperature superconductors), which demonstrates the presence of quantum mechanisms that overcome thermal decoherence.

Mathematically speaking, complex systems lie at the boundary between perfect order and fractal chaos, arising at “the edge of chaos” (Packard, 1988), which is sometimes referred to as a “poised realm” (Lewin, 1999) between stability and chaos. This delicate balance is rich in creativity and information storage potential, a region of phase space in which “chaos and stability pull in opposite directions” (Lewin, 1999). The boundary itself is fractal, which implies that the emergent properties of a system are highly sensitive to initial conditions. Also, because of the randomness in the system, inevitably, given enough time, there will be a movement out of that zone of criticality resulting in population collapse, a mass extinction event. In other words, precisely those conditions that make a system adaptive and “alive” are those that can eventually lead it to fall into either a too rigid form of order or conversely into chaos, resulting in an inability to adapt, i.e., death.

## Quenched disorder, measurement problems, and uncertainty across scales

The first three criteria constituting general features of complex systems—relationship of the size of the system's population and its diversity of interactions to increasing complexity, the predominance of negative over positive feedback loops, and the importance of local interactions within the system's holarchy—are straightforward to see across scales. However, the limited randomness in the system, sometimes termed “quenched disorder,” has different sources at different scales which relate to functional, scale-specific interactions.

Interactions between molecules are also sometimes governed by specific chemical processes, but when it comes to important biomolecules, there is a form of constrained randomness that particularly merits the phrase “quenched disorder.” Such interactions are driven by Brownian motion of aqueous solution (within cells, within and between tissues). The importance of Brownian motion as a source of the energy for movement and other physiologic processes was originally demonstrated by Yanagida in exploring the role of ATP in single pair interactions of actin and myosin filaments (Yanagida et al., 2000; Theise and Harris, 2006). The prevailing dogma prior to Yanagida's work assumed that the energy of movement of myosin across actin filaments within myocytes was provided by the binding of ATP to the myosin filament and subsequent hydrolysis explaining the stepwise motion of the myosin along the actin filament. However, Yanagida demonstrated that the energy of movement was provided by the Brownian motion induced by the kinetic energy of the water molecules bombarding the myosin molecule when anchored to the actin filament. The ATP did not propel the movement, it provided the precise amount of energy necessary to “quench” the Brownian disorder into directional movement. This is now recognized in the functions of many “molecular motors,” so that cells can be viewed as emergent properties arising from self-organizing biomolecules in water (Yanagida et al., 2007; Yanagida and Ishii, 2017). Interestingly, Einstein's paper on Brownian motion (Einstein, 1906) may now be considered foundational for understanding the dynamics of biological systems.

Considering cells at the next higher level of scale, we find that quenched disorder applies to other inter- and intracellular processes, all of which demonstrate restrained stochasticity. This concept was controversial among cell biologists who considered cell behaviors and differentiative fate paths to be largely deterministic (Morrison and Weissman, 1994). Nonetheless, other views were expressed. Lewontin (2000) had stated at the time, “the inside and the outside co-determine the cell”. More cell biologists recognized that cells have no inherent identity or behavior independent of the environment in which they find themselves. For example, any experimental procedure which isolated a cell for study would necessarily disturb that environment and therefore lead to changes in the nature of the cell's functions or even its differentiative fates (Theise, 2006; Theise and D'Inverno, 2004; Theise and Harris, 2006). With the simplest act of venipuncture to isolate cells from the blood to enzymatic digestion and mechanical dissociation to derive them from tissues, there is no way to avoid interaction that would inescapably lead to alterations in gene expression and, thus, changes in the nature of the cell. A biological equivalent of the quantum “measurement problem” could thus already be discerned. Studies in cell cloning and cell plasticity, definitively overturned notions of cellular stochasticity (Daley, 2012). One culminating demonstration of adult cell plasticity came with multi-organ plasticity deriving from a single, clonally explanted bone marrow cell (Krause et al., 2001). We can thus formulate a biological variation of Heisenberg's uncertainty principle: that any attempt to observe a cell seems *at least likely* to alter the nature of the cell at the time of observation. Indeed, if a cell is an active “observer” of its environment, as extensively argued by (Albrecht-Buehler 2005), it stands to reason that making experimental observations on cells must affect their activities.

These statements could be termed Heisenberg's “Cellular Uncertainty” (Theise and Harris, 2006; Potten and Loeffler, 1990; Theise and Krause, 2001, 2002), due to the exquisite sensitivity of a cell to any form of manipulation or identification. Just as Heisenberg's uncertainty principle in physics asserts the impossibility of measuring both position and momentum of a particle, actual experimental data indicate that cells could not be isolated and probed to definitively assert “what type of cell it is.” As for Heisenberg's uncertainty in physics, this limitation is inherent in the complex nature of biological reality (Theise and Harris, 2006; Theise, 2023; Kurakin, 2005a,b).

Studies of epigenetics in parallel with cell cloning and plasticity research show that all epigenetic mechanisms are flexibly changeable and therefore also stochastic, not deterministic as elaborated on in Davis et al. (1987), Xie et al. (2004), Collas (1998), Collas (2003), Bruniquel and Schwartz (2003), Cuthbert et al. (2004), Wang et al. (2004), and Shi et al. (2004), and summarized in Theise and Harris (2006). No gene activation or restriction is rigid and permanent.

Cellular Uncertainty indicates that the mechanism of evolution by random mutations is highly unlikely (Wagner, 2012). An alternative explanation proposed involves quantum search algorithms that can rapidly accelerate the process of optimization of the fitness function (McFadden, 2002), i.e., a limited rather than unrestricted randomness typical of complex systems. The 2012 Nobel prizes in Medicine and Physiology to J. Gurdon for cloning and S. Yamanaka for induced pluripotent stem cell technology were recognitions of this flexibility resulting not from pure randomness, but the quenched disorder of cellular epigenetics and gene-gene interactions (Daley, 2012).

Finally, a less well-known aspect of quantum biology, namely quantum metabolism, sheds additional light on Cellular Uncertainty. Living systems maintain far-from-thermodynamic-equilibrium conditions whereas non-living systems exist at thermodynamic equilibrium determined by minimum free energy conditions. Empirical rules relating metabolic rate and body size are called allometric laws of physiology (Agutter and Tuszynski, 2011) and were found to satisfy a mathematical relation which is non-linear. A quantum mechanical explanation of this phenomenon was developed by Demetrius (2006) and is based on the Einstein-Debye methodology of the quantum theory of specific heats of solids. Quantum metabolism is based on an analog of Planck's quantization principle whereby the metabolic energy generated by an enzymatic oscillator with frequency ω is quantized according to the following rule: *E*_*n*_ = *n*κω. Here, κ is the metabolic analog of Planck's constant referred to it as the biological Planck's constant. Quantization of metabolic energy is due to integer ATP numbers being produced in the cell's mitochondria. Quantum metabolism recapitulates the observed allometric scaling laws whose exponents depend on spatial dimensionality of the enzymatic network, d, and range from 1/2 to 2/3, to $\frac{3}{4}$, respectively, as d changes from 1, to 2, to 3 (Demetrius and Tuszynski, 2010).

Using the Heisenberg uncertainty principle for the rate of ATP production in cellular mitochondria, it was estimated that the biological equivalent of Planck's constant is on the order of κ = 2 × 10^−24^ J s, which is ~3 × 10^11^ times larger than Planck's physical quantum of action. While the physical Planck's constant corresponds to a single atom, the biological constant corresponds to the energy production cycle of the mitochondrion. With approximately 7 × 10^27^ atoms in the human body comprising some 3.6 × 10^13^ cells and some 1,000 mitochondria per cell, this gives 2 × 10^11^ atoms per mitochondrial “sphere of influence” within the cell. This provides a plausible explanation for the ratio between the biological and physical Planck constants, which i.e., κ/*h* = 3 × 10^11^.

Next, we can estimate the Planck quantum of action for microtubules, κ_*T*_, where the subscript “T” refers to the tubulin dimer. A characteristic energy unit here is that of GTP hydrolysis energy, i.e., approximately 3k_B_T or 10^−20^ J and a characteristic time scale for its turnover is 1 ns. Consequently, the quantum of action for microtubules is on the order of 10^−29^ Js, which is 2 × 10^5^ smaller than the value for mitochondria reflecting the differences in sizes of these structures.

We can also apply a similar analysis of the energy-time uncertainty relationship as for mitochondria and microtubules to neuronal dynamics. Let us consider ion channels in a single neuron that is firing (Kandel et al., 2013). Typical timescales involved are ~ msec, with an electric potential difference on the order of Δ*V*~ 50 mV, and an electric current on the order of ~1 picoamperes, which is equivalent to ~10^7^ ions/s. We find that the neuronal equivalent of the Planck constant, κ_*N*_, where the subscript “N” refers to the neuron, is on the order of ~10^−19^ J s. This value is 5 orders of magnitude greater than the biological (mitochondrial) Planck constant and 10 orders of magnitude greater than the one corresponding to microtubule dynamics.

Finally, we estimate the same quantity for the entire brain. Roy and Kafatos (1999) critically discussed the applicability of quantum formalism to brain dynamics including an anatomical perspective. They analyzed both time and energy scales in this context, both at a micro- and macro-scale. The physiological temperature, T = 300 K, translates into a thermal energy scale of approx. 25 meV, which corresponds to a time scale of 0.1 ps, clearly too short compared to cellular processes that operate on time scales of ns and above. In connection with the Heisenberg uncertainty principle, they remarked that a similar formula has been introduced to information theory (Gabor, 1946), stating that the product of a frequency uncertainty and time uncertainty is on the order of unity, namely:


$$\begin{array}{l} \delta \text{t}\delta \omega =½ \end{array}$$

Furthermore, they proposed an analogous property on the context of brain dynamics whereby

δtδω = b, where b is a pure number and denotes the “brain constant” which needs to be related to action quanta like in Planck's constant. Furthermore, according to Libet's (1985) experiments on the pre-processing time for activation of specific sites in the brain required to trigger artificial somatic sensations, relying on routine psychophysical procedures. The subjective experience of the conscious will to act was found to be preceded the action by only 200 ms, with a margin of error of approximately 100 ms which corresponds to δ t. To estimate the brain equivalent of the Planck constant, κ_*B*_, we note that the power consumption of the brain is typically 25 W, which corresponds to E/t. It is safe to assume that ΔP = ΔE/Δt is on the order of 0.1 W. Combining these numbers in a product we obtain an uncertainty relation for brain dynamics as:


$$\begin{array}{l} \Delta E\Delta t\;=\Delta P\;{\left(\Delta t\right)}^{2}=\;10^{-3}J.s\;=\kappa_{B} \end{array}$$

where the subscript “B” refers to the brain.

Above, we have estimated the “biological Planck constant” for a mitochondrion to be on the order of κ_*M*_ = 10^−24^ Js. The average size of a brain is 1.5 kg which can be compared to the size of a mitochondrial “sphere of influence” in the cell that involves 1.9 × 10^11^ atoms, and has a mass of approximately 10^−15^ kg. Therefore, we find that the ratio of the masses of the brain to that of the mitochondrial sphere of influence is M/m = 10^15^ with the ratio of the power outputs also:


$$\begin{array}{l} \text{P}/\text{p}=\text{1}0^{\text{15}} \end{array}$$

In summary, we have found that the various quanta of action relevant to the functioning of the several biological systems of interest to neuroscience are related to each other through the approximate ratios: κ_*B*:_ κ_*N*:_ κ_*M*:_ κ_*T*_: *(h/2*π*)/2* ~ 10^20^: 10^15^*:* 10^10^*:* 10^5^: 1, where Planck's quantum of action is taken as unity. These are orders of magnitude estimates that are based on typical spatial, energetical and temporal uncertainty estimates. It appears that each level of the emerging hierarchy is built on the foundation of a lower level with the quantum of action increasing by approximately 10^5^. This implies a progression of structural organization from atomic to protein-level, to organelle-level, to cell-level and eventually to an organ level as exemplified by the brain. This analysis can be extended further on the organizational scale of living systems.

Arguably, much of the structural organization of the human body can be described using a hierarchical description using the mathematics of multifractals (West and Goldberger, 1987). The collective self-organization of bodies into still larger scale structures—from colonies of ants, flocks of starlings, and bait balls of fish up to even larger communities such as cities, cultures, economies, and ecosystems—eventually takes us to the composite global ecosystem of the Gaia hypothesis (Lovelock, 2003; Lovelock and Margulis, 1974). It is self-evident that all these living systems are neither deterministic nor machine-like any more than are cells. The assumptions of constrained randomness are, in fact, derived from complexity modeling of all such systems (Theise, 2023). By this means, we can perhaps move from an understanding of Cellular Uncertainty to a generalized principle of Biological Uncertainty.

## Reconciling classical non-linear theories with quantum mechanics

In previous sections we have emphasized the importance of complexity theory in understanding the behavior of living systems. Complexity theory with its interesting characteristics related to chaos, fractality, and pattern formation, relies on non-linearity that characterizes the dynamics of these systems. Non-linearity, in turn, is a property of feedback loops generated by interactions between a system's units. As we have also emphasized above, living systems exhibit features that are best described by quantum physics, for example energy quantization, uncertainty, tunneling, superposition of states, entanglement, etc. This combination of classical non-linear dynamics and quantum mechanical features poses a major challenge because quantum mechanics is inherently a linear theory by virtue of the wave function superposition principle, a key postulate of quantum mechanics and the foundational equations, the Schrödinger and Dirac equations. A similar situation exists for quantum many-body systems such as those described by modern condensed matter physics.

Fortunately, a formal solution to this problem exists that applies exceedingly well to quantum systems close to criticality, i.e., under conditions close to bi- (or multi-) stability, which appears perfectly suited for biological systems. The mathematical formalism developed exquisitely for this type of situation is called the Method of Coherent Structures (MCS). Living systems represent metastable steady states with the absolutely stable state being the condition of death. While this paper's size and format is not suitable to present detailed mathematical description of the MCS approach, in the text below we outline this method's broad strokes that allows one to keep the non-linear features of the many-body system in question together with its emergent quantum properties. Both the details of the mathematical method used and its physical applications can be found in the monograph by Dixon et al. (1997). In a series of papers (Dixon and Tuszynski, 1989; Tuszynski and Dixon, 1989), the MCS was first developed to treat strongly interacting many-body systems of particles described using the generic effective second-quantized Hamiltonian.


$$\begin{array}{l} H_{eff}=\underset{k,l}{\sum }\omega_{k,l}{q_{k}}^{†}q_{l}+\underset{k,l,m}{\sum }\Delta_{k,l,m}{q_{k}}^{†}{q_{l}}^{†}q_{m}q_{k+l-m} \end{array}$$ (1)

Here, the first term describes one-body interactions (ω_*k, l*_ are the energy levels of individual elements of the system) and the second involves two-body exchanges of energy (i.e., Δ_*k, l, m*_ are the pair-wise interactions between the elements). The operators *q* and *q***+** are second quantized creators and annihilators of excited energy states of the systems' elements, respectively, which can satisfy either Bose-Einstein or Fermi-Dirac commutation relationships, hence have general applicability. The labels on the operators *k, l*, and *m* refer to the interacting quantum particles (or indeed units in a biological system) but the method developed does not rely on this identification and allows other physical representations, for example, linear or angular momenta, or spin variables. The dispersion relations for the coefficients ω and Δ describe coupling strengths between the interacting units. The range of applicability of this model is very broad and includes most phenomena in condensed matter physics (crystal lattice dynamics, magnetism, electrons in metals, superconductivity, superfluidity, atoms in chemical molecules), to name but a few of the examples worked out in detail (Dixon et al., 1997).

The next step is to derive Heisenberg equations of motion for the creation and annihilation operators


$$i\hbar \partial_{t}q_{\eta }=\sum_{k}\omega_{\eta ,k}q_{k}-\sum_{k,m}\left(\Delta_{k\eta m}-\Delta_{\eta km}\right)q_{k}^{†}q_{m}q_{k+m-\eta }$$ (2)

$$i\hbar \partial_{t}q_{\eta }^{†}=-\sum_{k}\omega_{\eta ,k}q_{k}^{†}+\sum_{k,m}(\Delta_{km\eta }-\Delta_{mk\eta })q_{k}^{†}q_{m}^{†}q_{k+m-\eta }$$ (3)

Then, by introducing a quantum field operator Ψ (and its Hermitian conjugate Ψ^+^) using standard rules of quantum field theory


$$\begin{array}{l} \psi \left(r\right)={\Omega^{-}}^{1/2}\underset{k}{\sum }exp\left(-ik\cdot r\right)q_{k} \end{array}$$ (4)

leads to the derivation of Heisenberg equations of motion for the quantum field operators taking the form:


$$\begin{array}{l} i\hbar \partial_{t}\psi =v_{0}\psi +iv_{1}\cdot \left(\nabla_{r}\psi \right){-}^{1}{/}_{2}\nabla_{r}^{2}\psi +\Omega f\left(\eta_{0},k_{0},m_{0}\right)\psi^{+}\psi \psi \end{array}$$ (5)

The next step in the MCS is of cardinal importance as it allows to separate a classical component of the field, η_0_, also referred to as an envelope field, from the quantum fluctuations represented by the operator Λ


$$\begin{array}{l} \psi =\eta_{0}+\Lambda \end{array}$$ (6)

The resultant sets of equations have been analyzed and solved under numerous conditions but in the simplest case the classical non-linear envelope field, η, leads to the cubic non-linear Schrödinger equation, with a stationary case given by


$$½\;\nabla_{r}^{2}\eta +F_{0}\eta^{3}=E_{0}\eta$$ (7)

whose solutions include extended elliptic propagating waves, localized kinks, and bump solitary waves, which are canonical examples of non-linear waves. Adding dissipative terms and forcing, as would be required for living system applications, would allow for the existence of chaotic solutions as well as fractality in the corresponding phase space, which is important for proper modeling of living systems as emphasized above in this paper. Importantly, numerous exact solutions of the non-linear Schrödinger equation (in 1D, 2D, and 3D) are available in the mathematical physics literature, especially based on the Lie symmetry group analysis (Dixon et al., 1992; Gagnon and Winternitz, 1988).

Moreover, quantum effects can be obtained, to a very good degree of approximation, by linearization about the classical envelope solutions, η_0_. Again, in the simplest case the resultant (linear) stationary Schrödinger equation for the quantum fluctuation Λ with an Eigen energy value *E*_0_ has been found as


$$\begin{array}{l} -½\nabla_{r}^{2}\Lambda +3F_{0}\eta_{0}^{2}\Lambda =E_{0}\Lambda \end{array}$$ (8)

Using localized classical envelope solutions for η_0_ gives rise to quantum solutions in the form of low-lying bounded excitations and a high-energy continuum of states. Periodic solutions to the classical field equation, on the other hand, lead to the formation of both allowed and forbidden bands of the quantum states. As stated above, this approach allows to keep the non-linear character of the classical field with its characteristic properties that depend on the specifics of the constituent interactions between the individual elements forming the system (e.g., enzymes in a mitochondrion or cells in a tissue), which may lead to chaos, fractality and localized, solitary wave solutions. The resultant quantum fluctuations contained by the classical envelope maintain such properties as superposition, tunneling and entanglement as desired by quantum biology. Finally, extensions of this method to systems composed of coupled quantum fields (e.g., such as those envisaged by Fröhlich, involving phonons and excitons) have been also developed (Dixon et al., 1998) as well as extensions including thermal effects, which are required in biological systems (Tuszynski and Dixon, 2000).

While the above references use the MCS to describe the emergence of non-linearity from quantum interactions between particles and quasi-particles in physical and chemical many-body systems, specific applications to biology still remain to be properly explored. Notably, recently described noise-induced quantum synchronization of entangled superconducting oscillations can serve as a blueprint for such phenomena, for example, in neuronal systems, as explored in Tao et al. (2025). A sole direct application of the MCS to biological systems describes the polymerization dynamics of microtubules (Rezania and Tuszynski, 2008a,b) and its results compare very favorably to the experimental data for microtubule assembly/disassembly phenomena.

We should emphasize, however, that in any biological system, there is no single category of *thing* which has inherent existence. All biological structures and properties that have an appearance of inherent existence at one level of scale are clearly merely phenomena which arise from interactions of lower scale agents (Theise and Kafatos, 2013). Their natures always manifest only at a specific, selected observational perspective. In turn, these ultimately, inescapably depend on the measurement issues familiar at the quantum level. While the MCS has only been applied so far to a single scale of organization, a nested description of the hierarchical organization of biological systems using the MCS is entirely possible.

Emergent self-organization—adaptive, self-sustaining, but prone to inevitable collapses – governs all such systems across all scales. Therefore, Biological Uncertainty, like Heisenberg's Physical Uncertainty, is inherent, not merely a technological limitation.

## Biological complementarity and beyond

This dependence on scale for the appearance of a biological entity as a thing vs. a phenomenon explicitly invokes a “complementarity” that is not dissimilar to that which pertains at the quantum level (Theise and Kafatos, 2013; Theise, 2023, 2005). No single description of an entity/process captures the entirety of the reality: waves vs. particles, things vs. phenomena. There is no element of a biological system that doesn't partake of the incompleteness, which seems similar to Gödelian incompleteness (Theise et al., 2024). Biological complementarity indicates:

“*that no single technique or perspective allows comprehensive viewing of all of a biological entity's complete qualities and behaviors; instead, complementary perspectives, necessarily and irrevocably excluding all others at the moment an experimental approach is selected, would be necessary to understand the whole. Systems biology and complexity theory reveal that, as in the quantum realm, experimental observations themselves limit our capacity to understand a biological system completely because of scale-dependent ‘horizons of knowledge' […] Specifically, observational selection is inherently, irreducibly coupled to observed biological systems as in the quantum realm. These nested systems, beginning with biomolecules in aqueous solution all the way up to the global ecosystem itself, are understood as a seamless whole operating simultaneously and complementarily at various levels.”* (Theise and Kafatos, 2013)

This correlates with Pauli's prediction that some objects would have a “many valued” set of complementary observable properties. The “horizons of knowledge” delineate the contingent boundaries between different scalar perspectives (Kafatos, 2009; Kafatos and Nassikas, 2011; Nadeau and Kafatos, 1999):

“*This selection of an observational stance is inseparable from descriptions of biology in accordance with views of thinkers such as von Neumann, Wigner, and Stapp that even at levels of scale governed by classical physics, at biological scales, observational choice remains inextricably woven into the establishment, in the observational moment, of the present conditions of existence.”* (Theise and Kafatos, 2013)

As a consequence, the study of cells isolated from a body *in vitro* are not the same cells as they were inside the body: a changed in environment means a changed cell. The cell membrane is not an unchanging structure that keeps the inside separate from the outside, far from it. All notions of boundaries dissolve from this perspective (Theise, 2023, 2005). Furthermore, concepts of symbiosis from Margulis (1981) are central to this aspect of biological behaviors (Margulis, 1981; Sagan, 2021). In developmental systems theory, the intimacy of environment and biological form are related reflecting these same principles (Oyama, 2000). Pattee stated that biological systems need both (rate dependent) structural and (rate independent) informational concepts for their description, suggesting that biological structures and information were a biological complementarity (Pattee, 1979a,b; Umerez, 2001). Kafatos and Nadeau (2000) demonstrate that most fields of study depend on complementarity frameworks. Similar concepts arise easily by relating biological networks of interacting agents to Markov blankets (Kirchhoff et al., 2018). Material engagement theory likewise explores such concepts (Malafouris, 2004).

## Scale/perspective dependence of boundaries—Heisenberg's cut

At the microscopic scale, our bodies are understood as communities of cells, the majority of which (>90%) do not carry “our own” DNA, but are mostly bacteria that make up our microbiomes, without which we cannot exist (Berg et al., 2020). At this cellular scale, our boundary extends significantly beyond our skin, through shedding of our skin cells from the surface and depositing bacteria on any surface we touch. Indeed, co-habitating humans and their pets comprise one single, unified microbiome that encompasses several large islands of otherwise distinct human and associated pet cells forming *holobionts* (Simon et al., 2019). At the cellular level, our boundaries are as wide as our physical environment and the other living beings with whom we share those spaces. At the molecular level our boundaries are wider still since our boundaries encompass the entire biomass of the planet. At the atomic level, our boundaries are as wide as the entire mass of the planet, organic *and* inorganic (Lovelock, 2003; Lovelock and Margulis, 1974).

If boundaries are scale/perspective dependent, then an answer to the puzzle of the Heisenberg-von Neumann cut becomes apparent (Atmanspacher, 1997; Stapp, 2017). For Heisenberg, this “cut” was the hypothetical boundary between infinitesimal levels of scale at which quantum events occur and the larger scales where classical physics and the separation of subject (experimenter) and object (e.g., Schrödinger's cat) occur. von Neumann asked where in the human body this cut *actually* exists, where the interface between physical phenomena involved in perception interface with how the mind manifests that perception (Narasimhan et al., 2019). But if there is no reified, inherent boundary between human subject and its object, if all boundaries are contingent, there can be no fixed “cut,” by Heisenberg's view or von Neumann's.

## Wave functions and Kauffman's “adjacent possibles”

Much debate in physics still surrounds the concept of the “collapse of the wave function.” Depending on the interpretation of QM, this collapse may be an actual event or merely a mathematical expression of a result that is unreflective of an actual underlying process. There seems to be an analogous aspect of biological systems.

The term “adjacent possibles” was coined by Kauffman for the range of *possible* changes that *might* arise within a complex system's emergent properties because of the quenched disorder of the system (Kauffman, 1995, 2019). The low-level random events in the system may give rise to changes in emergent properties that are neutral regarding the environment, adaptive, or destructive. Kauffman considered these changes, when adaptive, to be Darwin's “pre-adaptations” or Gould's “exaptations,” alterations that precede the environmental changes that drive natural selection. Other “possible” states may be non-adaptive and hence contribute to evolutionary development or even lead to extinction events (Ridley, 2004; Gould and Vrba, 1982).

The adjacent possibles thus generate a phase space around any current complex system. Every biological system in every instant, explores a wide array of “adjacent possible” changes to its emergent properties. It is only with the arrival of the next moment that the range of adjacent possibles becomes limited to the *actual* possible which we experience. We propose that this actual possible derives from the adjacent possibles in the way that, with a “collapse of the wave function,” the actual particle with its actual properties is recognized. In the quantum realm, it is the instance of observation that leads to collapse. In the complex biological realm, it is with each arrival of the present moment that collapse of the adjacent possibles into the actual occurs, perhaps with each moment of the living system's self-awareness (Theise, 2023).

## Connection to neuronal information processing

A possible connection between QM and neuroscience has been hypothesized to involve neuronal microtubules (MTs) (Stuart, 1998). Unlike other cells, neurons once formed, do not divide, and so neuronal MTs remain assembled in parallel bundles, providing a stable medium for information processing and memory encoding. In axons, MTs are organized in parallel to each other with the same polarity. However, MTs in dendrites are short and aligned in mixed polarity networks interconnected by microtubule-associated proteins. Due to their key roles in cell organization and functioning, and their regular lattice structure, MTs appear to be well-suited for information processing roles in the cell. In this context, each tubulin in an MT lattice could represent a particular conformational state, e.g., binary “bits” of either 1 or 0, which could interact with neighboring tubulin states by dipole coupling in a molecular “automata” algorithm in order to generate interactive patterns and hence process information. More recently, tubulin states were proposed to represent quantum bits (“qubits”) in quantum computational MT lattice networks which would offer unparalleled computational capabilities (Craddock et al., 2014). MTs have also been hypothesized as candidates for memory encoding in the brain (Craddock et al., 2012) although it is unclear if this would be a classical or quantum encoding process. The highly negative surface charge of MTs induces positively charged counter-ions in the cytoplasm to condense forming an ionic cloud around the filament. Consequently, electric fields generated by firing neurons can strongly affect the neural cytoskeleton leading to rapid inter- and intra-cellular communication. MTs can propagate ionic waves and make contact with actin filaments, which can then relay these waves all the way to ion channels. MTs whether as part of a quantum mediated function or a classical electromagnetic field driven system, may influence the cell's response to neurotransmitters via depolarization and neuronal firing. These mechanisms can connect groups of neurons and make them more or less active eventually effecting cognitive processes. The MT cytoskeleton in neurons appears to be a fertile area for potential interactions between classical and quantum effects in biology.

## Summary and conclusion

Complexity theory provides avenues for the exploration of revisions that may be required of biology in view of the insights offered by modern physics. Such revisions would include variations of the QM measurement problem and, ultimately, recognition that subject:object separation was only a useful fiction for reductive science. Biology may require precisely the same accommodations to the nature of “the real” as QM imposed on physics. The QM founders, in particular Bohr, Pauli, and Schrödinger, were several generations ahead of the biologists of the rest of the 20th century. It is in this 21st century that the contingent and complex natures of biological systems are only starting to be appreciated. Therefore, we argue that direct correlates of QM types of observational/measurement issues, such as Complementarity, Quantization and Uncertainty, can be recognized in our understanding of complex biological systems. Moreover, this analysis can provide biological correlates to such recognizable puzzles as the “collapse of the wave function” and the nature of the Heisenberg-von Neumann “cut.”

In this paper, we have provided a possible methodology for a consistent inclusion of both classical complexity and quantum physics using a mathematically sophisticated methodology (MCS) originally developed for and applied to many-body quantum condensed matter applications. We sketched the outlines of this approach which should find specific applications in the context of biological systems that are characterized by both the complexity of multi-agent non-linear dynamical structures and inherent uncertainties of their behaviors, which are consistent with quantum-like principles. Whether these properties of biological systems are of a different origin or order of meaning from those seen in QM, or, instead, arise directly from QM level effects as they scale upward into self-organizing biological systems, remains unsettled. The work of the coming generations is to assimilate these concepts into “orthodox” biology, much in the way that the work of assimilating QM into classical physics continues to the present day.

A more creative and robust understanding of biology will involve hierarchies—or “holarchies” (Theise and D'Inverno, 2004)—of systems that are not merely predictable machines, but “wholes” that are truly and unpredictably greater than the sum of their parts.

Finally, if quantum mechanics does indeed play a vital role in the fundamental mechanisms of life, then it follows that the disruption of quantum processes, for example, by extraneous electromagnetic fields, chemical contamination or by reversion to normal rapid decoherence, will imply serious consequences for the biological functionality of the cell, possibly triggering the onset of a pathological transformation.

## Data Availability

The original contributions presented in the study are included in the article/supplementary material, further inquiries can be directed to the corresponding author.
